# Meaningful Assessment or Minimum Compliance: PASRR for Nursing Home Residents with Mental Illness

**DOI:** 10.1093/geroni/igab046.3080

**Published:** 2021-12-17

**Authors:** Taylor Bucy, Kelly Moeller, John Bowblis, Tetyana Shippee

**Affiliations:** 1 University of Minnesota, University of Minnesota, Minnesota, United States; 2 Miami University, Oxford, Ohio, United States

## Abstract

The Omnibus Budget Reconciliation Act (OBRA) of 1987 included provisions for the Preadmission Screening and Resident Review (PASRR) program, which requires states to create and maintain systems to assess persons with serious mental illness (SMI) seeking NH care. The prevalence of SMI in NHs is increasing, and little is known about the effectiveness of the PASRR program intervention. We conducted 20 interviews with state and national PASRR stakeholders, including assessors, hospital discharge planners, mental health advocates, geriatricians and geriatric psychiatrists. Interview data were triangulated with state provided materials on PASRR collection and implementation. Based on these interviews, we identified four themes: 1) variation in the implementation of federal PASRR legislation across states and jurisdictions, 2) the need for investment in professional development and workforce capacity, 3) lack of usefulness of PASRR in ongoing care planning, and 4) the need to consider the role of age, race/ethnicity, and stigma on quality of care for NH residents with SMI. Stakeholders agree that PASRR legislation was well intentioned, but also expressed concern regarding the completion of PASRR as an issue of compliance versus meaningful assessment. More work is needed to determine how best to develop and support the care needs of people with SMI, while being mindful of the original goals of deinstitutionalization that prompted OBRA passage. In order to assess the impact of the PASRR program on quality of care and mental health outcomes, further research should take an evaluative approach through meaningful use of PASRR data.

